# Implementation of a virtual and in-person hybrid hospital-at-home model in two geographically separate regions utilizing a single command center: a descriptive cohort study

**DOI:** 10.1186/s12913-023-09144-w

**Published:** 2023-02-09

**Authors:** Margaret R. Paulson, Eliza P. Shulman, Ajani N. Dunn, Jacey R. Fazio, Elizabeth B. Habermann, Gautam V. Matcha, Rozalina G. McCoy, Ricardo J. Pagan, Michael J. Maniaci

**Affiliations:** 1 Division of Hospital Internal Medicine, Mayo Clinic Health System, Menomonie, WI USA; 2 Medically Home LLC, Boston, MA USA; 3 Administrative Operations, Mayo Clinic, Jacksonville, FL USA; 4 Health Care Delivery Research, Mayo Clinic, Rochester, MN USA; 5 Division of Hospital Internal Medicine, Mayo Clinic, 4500 San Pablo Rd. Jacksonville, Florida, Florida 32224 USA; 6 Division of Community Internal Medicine, Geriatrics, and Palliative Care, Mayo Clinic, Rochester, MN USA

**Keywords:** Home hospital, Hospital at home, Telehealth, Health care delivery, Health services research, Acute care, Mobile integrated healthcare, Virtual hybrid

## Abstract

**Background:**

As providers look to scale high-acuity care in the patient home setting, hospital-at-home is becoming more prevalent. The traditional model of hospital-at-home usually relies on care delivery by in-home providers, caring for patients in urban communities through academic medical centers. Our objective is to describe the process and outcomes of Mayo Clinic’s Advanced Care at Home (ACH) program, a hybrid virtual and in-person hospital-at-home model combining a single, virtual provider-staffed command center with a vendor-mediated in-person medical supply chain to simultaneously deliver care to patients living near an urban hospital-at-home command center and patients living in a rural region in a different US state and time zone.

**Methods:**

A descriptive, retrospective medical records review of all patients admitted to ACH between July 6, 2020, and December 31, 2021. Patients were admitted to ACH from an urban academic medical center in Florida and a rural community hospital in Wisconsin. We collected patient volumes, age, sex, race, ethnicity, insurance type, primary hospital diagnosis, 30-day mortality rate, in-program mortality, 30-day readmission rate, rate of return to hospital during acute phase, All Patient Refined-Diagnosis Related Groups (APR-DRG) Severity of Illness (SOI), and length of stay (LOS) in both the inpatient-equivalent acute phase and post-acute equivalent restorative phase.

**Results:**

Six hundred and eighty-six patients were admitted to the ACH program, 408 in Florida and 278 in Wisconsin. The most common diagnosis seen were infectious pneumonia (27.0%), septicemia / bacteremia (11.5%), congestive heart failure exacerbation (11.5%), and skin and soft tissue infections (6.3%). Median LOS in the acute phase was 3 days (IQR 2–5) and median stay in the restorative phase was 22 days (IQR 11–26). In-program mortality rate was 0% and 30-day mortality was 0.6%. The mean APR-DRG SOI was 2.9 (SD 0.79) and the 30-day readmission rate was 9.7%.

**Conclusions:**

The ACH hospital-at-home model was able to provide both high-acuity inpatient-level care and post-acute care to patients in their homes through a single command center to patients in urban and rural settings in two different geographical locations with favorable outcomes of low mortality and hospital readmissions.

**Supplementary Information:**

The online version contains supplementary material available at 10.1186/s12913-023-09144-w.

## Introduction

The modern concept of acute medical care in the home setting, commonly referred to as “hospital at home”, was first implemented over 25 years ago [1]. This healthcare delivery model was built upon the belief that patients would have both a superior experience as well as recover faster in the comfort of their home as opposed to a hospital or institutional setting. In this archetype of care, rather than admitting a patient to a medical facility for access to medical resources and monitoring, all necessary medical resources and appropriate level of monitoring are brought by the institution to the patient in their home. These resources include both in-person visits from physician, nursing, and ancillary clinical staff, as well as in-home care provided by the institution’s pharmacy, laboratory, radiology, and rehabilitative services.

Over the past decade, several institutions have attempted to further develop this model by including post-acute care within the episode as well as offering care to patient populations beyond typical medical patients [2–4]. Studies on these initial hospital-at-home programs have demonstrated that a more favorable patient experience can be achieved at home while simultaneously reducing direct hospital stay costs, 30-day readmissions, and 30-day mortality [5, 6]. The hospital-at-home model offers a unique opportunity to support patients along their continuum of illness and recovery.

The majority of hospital-at-home programs in the US have been centered in urban settings with care orchestrated through academic medical centers [1–6]. There is little literature on hospital-at-home implementations in rural regions of the US, already disproportionately affected by decreased access to healthcare [7, 8]. Rurality is on a population density spectrum with the US Census Bureau defining “urban” as more than 1000 persons per square mile with “rural” population density as less than 1000 persons per square mile [9]. While simulation of rural hospital-at-home care is described in the literature [10], practical implementations of larger scale rural US hospital-at-home programs remain undescribed. Advances in technology offer promise for rural hospital-at-home, yet questions remain about the undemonstrated capabilities to safely support hospital-at-home patients in rural regions through remote telehealth partners.

We describe the implementation of Advanced Care at Home (ACH), Mayo Clinic’s hybrid virtual and in-person hospital-at-home model that combines a single virtual physician-staffed command center with in-home advanced practice provider visits and a vendor-mediated in-person medical supply chain that can deliver care in two diverse settings, urban and rural. We hypothesize that the ACH model of hospital-at-home can use a centralized, single command center to coordinate and deliver inpatient-level care to high-acuity patients in two separate US regions while maintaining the high-level of safety and quality outcomes seen in previous hospital-at-home models.

## Methods

### Patient selection and setting

This study was approved by the Mayo Clinic Institutional Review Board as a descriptive, retrospective chart review under protocol number 20–010,753 and patient de-identified data was analyzed under protocol number 21–004,666. All methods were conducted in accordance with relevant guidelines and regulations. The study was conducted between July 6, 2020, and December 31, 2021, at two Mayo Clinic sites: Mayo Clinic in Florida, a 304-bed community academic hospital in Jacksonville, Florida, and Mayo Clinic Health System in Eau Claire, a 304-bed community hospital in Eau Claire, Wisconsin. US Census Bureau data from the 2020 census reported Jacksonville, FL in Duval County with a population density of 1,231 persons per square mile while Eau Claire, WI in Eau Claire County had a population density of 165 persons per square mile. The dates were selected to offer the largest data set available at the time of writing and to include both patients treated in ACH prior to the program’s participation in the Acute Hospital Care at Home waiver program, which occurred on December 4, 2020, and December 8, 2020, for Mayo Clinic in Florida and Mayo Clinic Health Systems Eau Claire, respectively, and those treated after the beginning of the waiver program. The inclusion criteria for this study were the following: 1) all patients admitted to the ACH program in Florida and Wisconsin with no age restrictions and 2) a medical diagnosis successfully treated in previous hospital at home models (decompensated heart failure, chronic obstructive pulmonary disease exacerbation, and urinary tract infection) [11–15] as well as any general medical or post-surgical condition our medical providers believed that we could safely treat in the home setting. Admission to the ACH program is completely voluntary. Patients provide both oral and written informed consent to participate in the ACH program. Patients must meet clinical and social stability criteria to ensure they can be safely cared for in the home. This includes a review of their clinical status and potential expected changes in their clinical picture to confirm the physician feels the patient is stable enough to receive inpatient care at home. The social stability confirms that a patient’s home is a safe environment to provide inpatient care, including confirming the presence of running water, electricity, etc. (Appendix A).

The operational hub of ACH, the Command Center, was established on the Mayo Clinic in Florida campus to oversee care of all patients within ACH in both Florida and Wisconsin. The ACH program combined three key components to provide on-demand acute medical care management in the home setting:

1. Command Center: Staffed with physicians, registered nurses (RNs), and advanced practice providers (APPs: nurse practitioners and physician assistants), who work alongside non-clinical service coordinators, linked together by technology to provide around-the-clock care to patients enrolled in the program across all sites.
2. Technology in the Home: Custom technology kits, including biometric devices for monitoring vital signs (Bluetooth-connected sphygmomanometer, thermometer, pulse oximeter, and a floor scale), a custom-configured tablet with video visit capability, a telephone to facilitate 2-way communication, a backup power supply, a backup cellular communication cradle-point, and an emergency response system bracelet to keep patients and their families connected to the care team.
3. Care Delivery Services: The model includes a full suite of care services, including APPs, community paramedics, RNs, aides, rehabilitative services, infusion therapy, phlebotomists, and basic radiography technicians dispatched to the patients’ homes to allow for the provision of scheduled and acutely activated urgent patient care needs.

The ACH program components are linked together by Medically Home Group's Cesia Continuum™ software platform, connecting the three elements of the model, and enabling the delivery of acute care services in the home.

### ACH Model of care

Once it is determined that a patient is eligible for ACH and consents to care, the ACH provider team receives a clinical handoff from the patient’s current provider, either the ED physician in the case of a hospital substitution patient, or the brick-and-mortar provider in the case of a reduced length of stay patient. Hospital Substitution patients are those that are admitted to ACH after an evaluation in the Emergency Department and a determination that the patient requires an inpatient admission. Reduced length of stay patients are those that have had at least one midnight in the physical hospital and have been determined as clinically appropriate to move their inpatient care home and continue their inpatient admission with ACH. An admission huddle is coordinated by the ACH physician, the ACH RN, and the service coordinator, or scheduler, to plan patient-specific care and address immediate care coordination needs. The patient is then physically transported home by community paramedics who also set and orient the patient and family to the in-home technology kit. The community paramedic continues facilitation of the admission visit performing a home safety assessment, history of home medications, administration of medications, and assisting the ACH hospitalist with the physical exam.

During the acute phase, daily rounds are conducted by video with the ACH physician. As part of the vendor-mediated in-person medical supply chain, the following services are provided in the home. In-home rounds are conducted by APPs on ACH day 1 and as needed if a patient has a change in condition. All acute phase ACH patients are seen twice daily by either an RN or a community paramedic overseen by the command center virtual RN. Basic radiographic exams, such as x-rays and ultrasound, are performed in the home. Any advanced imaging or procedures are accommodated by bringing the patient to the brick and mortar hospital and then returning them to their home upon completion. Labs are obtained and intravenous medications are administered by a visiting nurse or community paramedic. A few additional components of this vendor-mediated in-person medical supply chain include delivery of condition-specific meals, delivery of supplies, and removal of biomedical waste.

In the event of acute patient deterioration, the patient is immediately connected with the Command Center, and a virtual evaluation is performed. Depending on the acuity of the situation, the Command Center either activates 911 (a three-digit telephone number in the United States which can be used to connect any member of the American public directly with a Public Safety Answering Point dispatcher who can immediately direct emergency services such as police, fire and rescue, and emergency ambulance services) or immediately deploys a community paramedic to the patient’s home. The patient is evaluated while communicating with the Command Center for further guidance. Diagnostic capabilities on scene include 12-lead ECG and a handheld blood analyzer (iSTAT) which can rapidly perform point-of-care laboratory studies like hemoglobin and blood chemistry levels. Community paramedics have access to a fully equipped advanced life support ambulance and can manage the patient as appropriate.

**Fig. 1:**
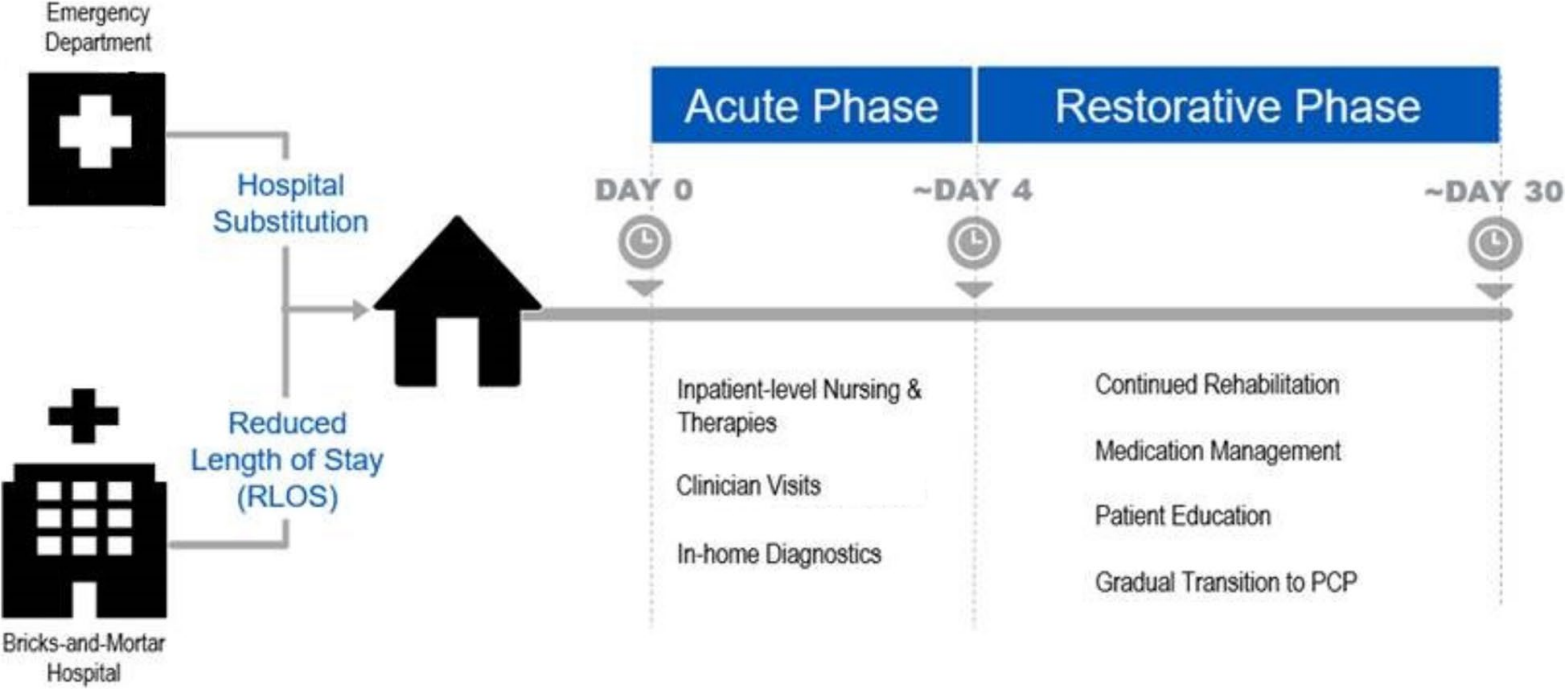
The Advanced Care at Home Model of Care

When the acute phase patient reaches a clinical stability level equivalent to that of discharge from the brick and mortar hospital, the patient is discharged from the inpatient encounter and then had the option to enter the ACH restorative phase, which consists of up to 30 days of post-acute virtual outpatient observation by the command center team. All patients were offered and encouraged to participate in the restorative phase that focused on patient-specific goals of care, including optimizing any medical and non-medical concerns that may be active at the time (whether related to the hospitalization or not) and monitoring for early signs of clinical decompensation. Goals and endpoints set for this phase include- but are not limited to- patient and family education, medication adherence, advanced care planning, and physical and occupational therapy. ACH APPs perform as-needed video and in-home visits and coordinate care during this phase of care. During the restorative phase, medications are prescribed and sent to the patient’s preferred pharmacy. Any recommended outpatient clinical appointments are coordinated and facilitated to optimize the patient’s medical conditions. The ACH providers work closely with the patient’s primary care team and any other provider’s the patient previously saw on a routine basis prior to their hospitalization with ACH. The combined acute and restorative phases of care have an estimated length of stay of 30 days** (Fig. ****1****)**, influenced by Federman et al. (2018), who found the 30-day bundle reduced hospital readmissions, emergency department visits, and admissions to skilled nursing facilities. [16] Near the end of the restorative phase, discharge from ACH is coordinated by the APPs, and a handoff is provided to the patient’s primary care provider. Home technology and equipment are removed upon discharge, and the patient is given discharge instructions prepared by the ACH providers.

### Data collection and analysis

Demographic and clinical data collected included patient volumes, sex, race, ethnicity, patient insurance identity, primary medical diagnosis, 30-day mortality rate (from date of acute phase discharge), in-program mortality, 30-day readmission rate and 30-day rate of outpatient Emergency Department (ED) visits (days 1–30 after acute phase discharge), rate of return to hospital during acute phase (outpatient ED or transfer back to inpatient), All Patients Defined Diagnosis Related Groups (APR-DRG) Severity of Illness (SOI), and patient length of stay in all phases of program. Primary medical diagnosis was first grouped into Clinical Classification Software for ICD-10-CM Diagnosis groupings and then subcategorized based on common disease diagnosis [17].. Rates of readmissions and outpatient ED visits within 30 days following discharge from acute ACH were limited to ACH admissions to the acute phase, while ACH admissions to the restorative phase only were excluded from these metrics; escalations from the ACH acute phase back to brick-and-mortar were counted as an escalation of care during the acute phase and not as a readmission. SOI was defined by the APR-DRG system subclass that is used by many United States hospital systems and uses a scale that ranges from 1 to 4, with 1 equaling “Low”, 2 equaling “Moderate”, 3 equaling “High” and 4 equaling "Extreme" [18]. Study data were collected and managed using electronic data capture tools hosted at Mayo Clinic. Patient demographics, clinical factors, and outcomes were summarized as *N* (%) for discrete factors and as median (IQR) and mean (SD) for continuous factors.

## Results

Between July 6, 2020, and December 31, 2021, there were a total of 686 admissions to the ACH program from 621distinct patients: 408 admissions in Florida and 278 admissions in Wisconsin. Of the 686 ACH admissions, 679 (99.0%) included the acute ACH phase while 7 (1.0%) included the restorative phase only. Of the 679 admitted to the acute phase, 543 (80.0%) chose to continue into the restorative phase of care following the acute phase. Overall, across admissions, 375 (54.7%) were men, 625 (91.1%) were white, 648 (94.5%) identified as not Hispanic or Latino, and 521 (76.0%) were government-insured patients. **(TABLE **1**).**

**Table 1:** Patient Demographics

	**Florida** ***n* = 408**	**Wisconsin** ***n* = 278**	**Total** ***n* = 686**
ACUTE / RESTORATIVE PHASES, n(%)			
Acute + Restorative	326 (79.9%)	217 (78.1%)	543 (79.2%)
Acute only	80 (19.6%)	56 (20.1%)	136 (19.8%)
Restorative only	2 (0.5%)	5 (1.8%)	7 (1.0%)
SEX, *n*(%)			
Male	220 (53.9%)	155 (55.8%)	375 (54.7%)
Female	188 (46.1%)	123 (44.2%)	311 (45.3%)
RACE, *n*(%)			
African American	7 (1.7%)	0 (0.0%)	7 (1.0%)
American Indian/Alaskan Native	2 (0.5%)	1 (0.4%)	3 (0.4%)
American born African	1 (0.2%)	0 (0.0%)	1 (0.1%)
Asian	1 (0.2%)	1 (0.4%)	2 (0.3%)
Asian Cambodian	1 (0.2%)	0 (0.0%)	1 (0.1%)
Asian Filipino	11 (2.7%)	0 (0.0%)	11 (1.6%)
Asian Indian	1 (0.2%)	0 (0.0%)	1 (0.1%)
Asian Korean	1 (0.2%)	0 (0.0%)	1 (0.1%)
Black or African American	17 (4.2%)	0 (0.0%)	17 (2.5%)
Choose Not to Disclose	6 (1.5%)	2 (0.7%)	8 (1.2%)
Other	9 (2.2%)	0 (0.0%)	9 (1.3%)
White	351 (86.0%)	274 (98.6%)	625 (91.1%)
ETHNICITY, n(%)			
Central American	0 (0.0%)	2 (0.7%)	2 (0.3%)
Choose Not to Disclose	10 (2.5%)	5 (1.8%)	15 (2.2%)
Hispanic or Latino	11 (2.7%)	2 (0.7%)	13 (1.9%)
Not Hispanic or Latino	380 (93.1%)	268 (96.4%)	648 (94.5%)
Other Spanish culture (except Spain)	3 (0.7%)	0 (0.0%)	3 (0.4%)
Puerto Rican	4 (1.0%)	0 (0.0%)	4 (0.6%)
Unknown	0 (0.0%)	1 (0.4%)	1 (0.1%)
INSURANCE, n(%)			
Commercial	86 (21.1%)	77 (27.7%)	163 (23.8%)
Government	321 (78.7%)	200 (71.9%)	521 (76.0%)
Self-Pay	1 (0.2%)	1 (3.6%)	2 (0.3%)

Ninety ICD-10-CM diagnoses were collected by the Clinical Classification Software and these were divided into 10 main categories, each having several diagnosis subcategories. The most common diagnosis seen were infectious pneumonia (27.0%), septicemia / bacteremia (11.5%), congestive heart failure exacerbation (11.5%), and skin and soft tissue infections (6.3%). **(TABLE **2**).**

**Table 2:** Patient Diagnosis

	**Florida** ***n* = 408**	**Wisconsin** ***n* = 278**	**Total** ***n* = 686**
**INFECTION**	218 (53.4%)	158 (56.8%)	376 (54.8%)
Pneumonia	96 (23.5%)	89 (32.0%)	185 (27.0%)
Septicemia / Bacteremia Complicated	60 (14.7%)	19 (6.8%)	79 (11.5%)
Skin / Soft Tissue infection	24 (5.9%)	19 (6.8%)	43 (6.3%)
UTI or pyelonephritis	14 (3.4%)	14 (5.0%)	28 (4.1%)
Infective Arthritis or Osteomyelitis	7 (1.7%)	10 (3.6%)	17 (2.5%)
Gastroenteritis / Intestinal Infection	11 (2.7%)	2 (0.7%)	13 (1.9%)
Peritonitis and intra-abdominal abscess	4 (1.0%)	4 (1.4%)	8 (1.2%)
Other infection	2 (0.5%)	1 (0.4%)	3 (0.5%)
**CARDIOVASCULAR DISEASE**	46 (11.3%)	45 (16.2%)	91 (13.3%)
Heart Failure	38 (9.3%)	41 (14.7%)	79 (11.5%)
Cardiac Dysrhythmia	3 (0.7%)	1 (0.4%)	4 (0.6%)
Circulatory disease	3 (0.7%)	1 (0.4%)	4 (0.6%)
Other Cardiac	2 (0.5%)	2 (0.7%)	4 (0.6%)
**HEMOTOLOGIC AND ONCOLOGIC DISEASE**	13 (3.2%)	4 (1.4%)	17 (2.5%)
Cancer / Neoplastic	5 (1.2%)	4 (1.4%)	9 (1.3%)
Venous Thromboembolism	5 (1.2%)	0 (0.0%)	5 (0.7%)
Anemia / Neutropenia	3 (0.7%)	0 (0.0%)	3 (0.4%)
**AIRWAY DISEASE**	23 (5.6%)	9 (3.2%)	32 (4.7%)
COPD	9 (2.2%)	4 (1.4%)	13 (1.9%)
Aspiration pneumonitis	5 (1.2%)	3 (1.1%)	8 (1.2%)
Respiratory failure	2 (0.5%)	1 (0.4%)	3 (0.4%)
Bronchitis or sinusitis	1 (0.2%)	1 (0.4%)	2 (0.3%)
Other Airway Disease	6 (1.5%)	0 (0.0%)	6 (0.9%)
**SURGICAL DIAGNOSIS**	27 (6.6%)	26 (9.4%)	53 (7.7%)
Complication after surgery	9 (2.2%)	15 (5.4%)	24 (3.5%)
Complication of a device, implant, or graft	6 (1.5%)	11 (4.0%)	17 (2.5%)
Complication of Transplanted Tissue	12 (2.9%)	0 (0.0%)	12 (1.8%)
**GASTROINTESTINAL AND HEPATOBILIARY DISEASE**	30 (7.4%)	6 (2.2%)	36 (5.2%)
Biliary tract disease	7 (1.7%)	3 (1.1%)	10 (1.5%)
Diverticulosis and diverticulitis	6 (1.5%)	0 (0.0%)	6 (0.9%)
Oral and esophageal	3 (0.7%)	1 (0.4%)	4 (0.6%)
Intestinal obstruction or ileus	2 (0.5%)	1 (0.4%)	3 (0.4%)
Inflammatory Bowel Disease	2 (0.5%)	0 (0.0%)	2 (0.3%)
Other gastrointestinal and hepatobiliary	10 (2.5%)	1 (0.4%)	11 (1.6%)
**KIDNEY AND UROLOGIC DISEASE**	30 (7.4%)	3 (1.1%)	33 (4.8%)
Acute renal failure	20 (4.9%)	1 (0.4%)	21 (3.1%)
Chronic kidney disease complication	5 (1.2%)	1 (0.4%)	6 (0.9%)
Fluid and electrolyte disorders	3 (0.7%)	1 (0.4%)	4 (0.6%)
Other Kidney and Urologic	2 (0.5%)	0 (0.0%)	2 (0.3%)
**MUSCULOSKELETAL DISEASE**	5 (1.2%)	11 (4.0%)	16 (2.3%)
Osteoarthritis	0 (0.0%)	6 (2.2%)	6 (0.9%)
Pressure ulcer of skin	1 (0.2%)	2 (0.7%)	3 (0.4%)
Other Musculoskeletal	4 (1.0%)	3 (1.1%)	7 (1.0%)
**ENDOCRINE DISEASE**	7 (1.7%)	9 (3.2%)	16 (2.3%)
Diabetes mellitus with complication	5 (1.2%)	8 (2.9%)	13 (1.9%)
Pituitary disorders	2 (0.5%)	1 (0.4%)	3 (0.4%)
**OTHER / MISCELLANEOUS**	9 (2.2%)	7 (2.5%)	16 (2.3%)

No patients died while in either the acute or restorative phases of the ACH program. Four patients (0.58%) died within thirty days of discharge from the acute phase of the ACH program; these 4 patients either had a short restorative phase (< 10 days) or no restorative phase and then were fully discharged from the ACH program. Two patients were readmitted to the hospital within 30 days where they passed shortly after hospital readmission, and the other 2 patients were enrolled into hospice care after ACH discharge where they passed away shortly after. Nineteen patients (2.8%) had an event in the acute phase of care that required escalation of care back to the brick-and-mortar hospital; of these, 5 only required care in the brick-and-mortar hospital for < 24 h, receiving an ED/hospital intervention and returning home within 24 h for continued ACH acute phase care and 14 required care and remained in the physical hospital for ≥ 24 h. After discharge from the ACH acute phase 37 patients (5.4%) had an ED visit without hospital readmission within 30 days and an additional 66 patients (9.7%) were readmitted to the hospital within 30 days. The median severity of illness was 3 (IQR 2–3) with a mean of 2.9 (SD 0.79). Median LOS was 3 days (IQR 2–5) during the acute phase of care. Patients who elected to enter the restorative phase of care (*n* = 550) had a median LOS in the restorative phase of 22 days (IQR 11–26). The median LOS over both phases of care was 22 days (IQR 7–29) for all patients. **(TABLE **3**).**

**Table 3:** Patient Outcomes

**Data Element**	**Florida (*n* = 408)**	**Wisconsin (*n* = 278)**	**Total (*n* = 686)**
Mortality Rate (30-day) from Acute Phase discharge^†^	3 (0.7%)	1 (0.4%)	4 (0.6%)
Mortality (in-Program)	0%	0%	0%
Escalation back to Hospital during Acute Phase^†^	9 (2.2%)	10 (3.7%)	19 (2.8%)
Stabilized and returned home within 24 h for continued acute ACH	2 (0.5%)	3 (1.1%)	5 (07%)
Remained in physical hospital for ≥ 24 h	7 (1.7%)	7 (2.6%)	14 (2.1%)
30-Day Emergency Department Visit, No Readmission*^†^	20 (4.9%)	17 (6.2%)	37 (5.4%)
Readmission Rate (30-day)*^†^	39 (9.6%)	27 (9.9%)	66 (9.7%)
Severity of Illness			
Mean (SD)	3.0 (0.80)	2.8 (0.77)	2.9 (0.79)
Median (IQR)	3.0 (3.0, 4.0)	3.0 (2.0, 3.0)	3.0 (2.0, 3.0)
Length of Stay, Acute Phase (days)			
N	406	273	679
Mean (SD)	3.9 (3.11)	3.9 (2.69)	3.9 (2.94)
Median (IQR)	3.0 (2.0, 5.0)	3.0 (2.0, 5.0)	3.0 (2.0, 5.0)
Length of Stay, Restorative Phase (days)^††^			
N	328	222	550
Mean (SD)	18.6 (9.29)	19.1 (8.23)	18.8 (8.87)
Median (IQR)	22.0 (10.0, 26.0)	22.0 (12.0, 26.0)	22.0 (11.0, 26.0)
Length of Stay, Total Both Phases (days)			
N	408	278	686
Mean (SD)	18.8 (11.36)	19.1 (11.12)	19.0 (11.26)
Median (IQR)	21.0 (7.0, 29.0)	23.0 (7.0, 30.0)	22.0 (7.0, 29.0)

Values displayed in the table are *N* (%) unless otherwise noted. SD: standard deviation, IQR: interquartile range

^*^30-days from time of Acute Phase discharge

^†^Among the *N* = 679 patients with an acute phase (*N* = 406 in Florida and *N* = 273 in Wisconsin)

^††^Among the *N* = 550 patients with a restorative phase (*N* = 328 in Florida and *N* = 222 in Wisconsin)

## Discussion

Our goal was to give a detailed description of a high-acuity hospital at home model with a single command center simultaneously supporting urban and rural regions in two different hospital settings in two states, across two time zones. Data from this hospital-at-home implementation demonstrate positive patient outcomes with high patient volumes, particularly noteworthy for a rural setting. We accomplished this with a new model of care that combined a virtual physician and bedside nurse with a software-controlled, vendor-mediated in-person medical supply chain.

Since the hospital at home model relies on acute care resources from a providing hospital, urban landscapes and academic centers have been the focus of previous hospital at home programs, leaving rural and under-resourced areas without the means to implement this model of care [2]. We wanted to devise a model that could be replicated in other hospital settings beyond our health system, while also having a broader outreach so this provision is not as constrained by geography or local resource availability. With the command center located in Florida, we were able to treat patients in two fundamentally different environments yet maintain patient volumes and outcomes such as LOS, SOI, and mortality among both sites. This centralized command center model, like some other hospital programs around the country, allows hospital at home care to be provided to patients in multiple geographic regions.

With our hospital at home model, we were able to go beyond typical diagnoses seen in home hospital models [11–15] and treat increasingly acute patients in their homes, such as bone marrow transplant patients on day-1 following transplant. These patients, who typically spend 14 or more days in the physical hospital, were able to recover primarily at home while receiving inpatient services. This shows the dynamic ability of a high-acuity focused model to meet the needs of many different types of hospitalized patients. Expansion of this model could move 25–30% of core diagnoses into the home setting [19]. ACH has a flexible chassis that allows care for a wide range of medical and surgical (post-operative) conditions. ACH was able to respond to specific needs at each Mayo Clinic location. For example, during a surge in COVID-19 cases in Eau Claire, Wisconsin, ACH was able to work with surgical teams to identify elective surgery patients eligible for in-home post-operative care rather than postponing surgery due to limited hospital capacity. The ACH model can adapt to meet situational institutional needs, while simultaneously meeting the needs of patients at those institutions, offering patients in rural and under-resourced regions desperately needed access to healthcare.

Our outcomes for the ACH hospital at home model were very encouraging. We found that 30-day mortality rates were quite low (0.58%) and we had no patients pass away while enrolled in the ACH program despite the mean severity of illness score of 2.9 across both sites. It should be noted that the low mortality rate both in the program and 30-days following the program could be attributed to the careful patient selection along both clinical and social parameters to ensure the patient would be safe at home and was stable enough to warrant receiving care at home. Interestingly, 80% of patients that participated in the acute phase of care chose to continue into the restorative phase of care. This optional phase of care was offered to patients as a mechanism to continue to stay connected to them and assist the patient using the supplier network of resources in the community in the event the patient began to decompensate. We suspect this high proportion of patients that chose to participate in the restorative phase could be reflective of patients’ desire to have an ongoing connection to their healthcare team along their healthcare journey. Compared to a reported 30-day mortality rate of traditional hospitalized patients discharged home with home health of 2.3% [20], our model shows promise of being at least as safe, and may even show the mortality reduction like previous traditional hospital-at-home programs [5, 21].

We also found that our readmission rates remain considerably low when compared to national averages [22]. This lower readmission rate could be attributed to our ability to escalate care in the home during the restorative phase of care, reducing the need to have the patient return to an emergency department. However, this could also be a direct reflection of the strict patient selection process, including the clinical and social stability screenings, that specifically look for acute but stable patients that will be safe in their homes.

There are several advantages to a virtual hybrid model of care, like ACH and several other home hospital models. First, all physician visits are virtual and utilize a single hospitalist for patient rounding at multiple geographic sites. A physician in our model can care for up to 15 acute patients and 30 restorative patients (or a combination of both). Nursing care for patient check-ins, medication administration, vital sign collection, and care plan communication is conducted by the virtual RNs at a ratio of up to 5 acute patients and 15 restorative patients (or a combination of both) to one virtual RN. This physician and RN virtual care is supplemented by frequent in-person touchpoints by clinical support staff local to each region. This typically includes two in-person nursing or community paramedic visits per day during the acute phase based on the Centers for Medicare & Medicaid Services Acute Care at Home waiver guidelines and state regulations. An APP local to the region also typically sees the patient each day in their home during the acute phase. Due to the travel time required between home visits, these APPs typically manage the care of up to 8 patients. This combination of virtual and in-person care diverges from previously established hospital at home programs prior to the introduction of the Acute Care at Home waiver program in the United States, in which all healthcare providers, including physicians, travel to the home [2, 23, 24]. The belief is that the ACH model, and others like it, allow the highest-cost providers to manage a larger volume of patients by removing the need for travel to the patient’s home. The patient volumes seen by providers are greater than what was previously reported in other hospital-at-home models [6, 11]. These findings may lead to this model being more affordable than traditional inpatient care or previous hospital-at-home programs. Additionally, there are significantly less fixed costs in operating a home hospital program than a physical hospital would incur. However, much ongoing cost analysis is needed to more fully understand the return on investment of programs like this and the impact to payers and patients.

A second advantage to this type of home hospital model, used by Mayo Clinic and others around the country, is the ability to recruit non-hospital owned resources in the form of multiple community medical vendors and use them to physically deliver health care in the home. This hub-and-spoke mechanism of the home hospital model used at Mayo Clinic promotes both safety and scalability. The vendor-mediated in-person medical supply chain can be established separately from the central institution housing the command center, covering a much larger geographic area or even different cities, as seen in ACH. Also, having multiple overlapping in-home vendor providers adds to the ability to get to multiple patients quickly, enhancing care delivery and adding a response-time safety net. A home hospital model developed using only internal resources to provide all in-home care may not offer this same advantage of redundancy. Our two sites had different approaches to provision of in-home services; specifically, the Florida program outsourced many of the services delivered in the home by partnering with third party vendors, while the Wisconsin site insourced many services. This variation was due to differential availability of services in urban versus rural environments, state regulations regarding in-home care, as well as existing external resources (e.g., home health agencies and pharmacy services). However, this outsource/insource pattern was not exclusively an urban/rural dichotomy; the configuration of Mayo Clinic’s internal resources at each site was also considered when making this decision. The divergence in provision of in-home services offered Mayo Clinic experience in delivering acute care at home under varying operational models, demonstrating the home hospital model’s flexibility and adaptability to local needs, resources, and regulations.

Finally, the virtual physician capability of this program creates a mechanism to potentially transfer a patient from one group of virtual providers to a separate group of more specialized virtual providers. For example, a rural physician could transfer oversight of their home hospital care to a physician more commonly staffed at a tertiary care center that may have more subspecialized expertise than the original provider, all while the patient remains at home in the home hospital model of care and they do not need to physically transfer to a different location for more specialized care. This ability to conduct a virtual “hospital to hospital transfer” would provide tertiary care centers the ability to give advanced care to isolated patients, limit resources used for physically transferring patients, and provide the convenience of having the patient stay at home while receiving multiple different levels of care.

### Limitations

There are several limitations in our study. First, the data was collected retrospectively from the electronic medical record. The inclusion criteria used to enroll patients in the program were broad, may be hard to replicate, and may have introduced inherent bias as the patients were screened for clinical and social stability prior to enrollment. The demographics of enrolled patients in Northeast Florida and Northwest Wisconsin may limit generalizability due to a predominance of white, non-Hispanic participants. Outcome data like mortality and readmissions may have inherent bias from screening and patient selection prior to enrollment. Mortality and readmission rate were non-matched to a brick-and-mortar inpatient unit. Additionally, outcome data may not be fully complete given that the data source is the electronic medical record and there may be patients that received care at external organizations that do not share readmission or mortality information with Mayo Clinic’s electronic medical record through the Care Everywhere platform.

## Conclusions

The Advanced Care at Home model delivers high-acuity, inpatient-level care at home to patients with a high severity of illness score. ACH simultaneously supports patients in both urban and rural sites with care overseen by a single telehealth medical command center, diverging from previously reported models in urban, academic centers. Patient outcomes of both sites were similar, particularly relevant for rural hospitals facing challenges in supporting hospitalized patients in their communities. The ACH program takes lessons from previously published hospital-at-home studies while fine-tuning the model to expand access to care by connecting hospitalized patients in their homes to healthcare team members in a virtual-staffed command center. Further studies are needed to determine “best practices” in the hybrid virtual hospital-at-home model and limitations of telehealth command centers.

## Supplementary Information

**Additional file 1: Appendix A.** Clinical Stability and Social Stability Screening Tool.

## Data Availability

The Datasets used for this study are located on secured servers within Mayo Clinic that are only accessible by Mayo Clinic staff to protect any identified or deidentified patient information. Deidentified datasets used and/or analyzed during this study are available from the corresponding author on reasonable request.

## Abbreviations

ACH: Advanced Care at Home
RNs: Registered nurses
APPs: Advanced practice providers
ED: Emergency department
SOI: Severity of Illness
APR-DRG: All Patient Refined-Diagnosis Related Groups
