# Supplementary material for: Implementation of a virtual and in-person hybrid hospital-at-home model in two geographically separate regions utilizing a single command center: a descriptive cohort study
Source: BMC Health Serv Res. 2023 Feb 9;23:139. doi: 10.1186/s12913-023-09144-w (PMC9911182; doi:10.1186/s12913-023-09144-w)
Supplement: Supplementary file 1 — Additional file 1: Appendix A. Clinical Stability and Social Stability Screening Tool. [file 12913_2023_9144_MOESM1_ESM.pdf]

Appendix A: Clinical Stability and Social Stability Screening Tool

Clinical Stability Screen

Must be NO:	Yes	No	Comment
Low diagnostic certainty requiring advanced diagnostics that cannot be performed in the home<br><br>If yes, select reason (if "other", select and describe):<br><br>☐ Active live telemetry monitoring<br>☐ Advanced imaging required (CT, MRI, nuclear stress)<br>☐ Cardiac catheterization required<br>☐ EGD/Colonoscopy required<br>☐ Lab monitoring not amenable to home (troponin, >q12 labs)<br>☐<br>Other____			
Higher acuity medical services may be required making admission to home clinically unsafe<br><br>If yes, select reason (if "other", select and describe):<br><br>☐ Intensive care unit required (i.e. vitals checked >q4hr, BG checked >q2hr, active drip titration etc.)<br>☐ Intubation risk elevated (i.e. history of intubation for respiratory conditions)<br>☐ New tracheostomy or mechanical ventilation required<br>☐ Vasopressor risk elevated (i.e. reasonable risk of fluid refractory hypotension)<br>☐ Unstable arrhythmias<br>☐ Elevated risk of respiratory compromise increasing risk of needing support with noninvasive positive pressure ventilation or intubation<br>☐ Home IV access limitations<br>☐<br>Other____			
Specific services required that cannot be Provided in the Home<br><br>If yes, select reason (if "other", select and describe):<br><br>☐ Risk for inability to obtain IV access<br>☐ Daily involvement of in person specialty consultation<br>☐ Blood transfusions likely to be needed (i.e. active GI bleed)<br>☐ Hemodialysis likely to be required (i.e. already on HD or newly anuric/severe ATN)			

☐ Respiratory isolation/airborne precautions (high TB concern)<br>☐ Pain such that would require adjustment of opiates or need for IV therapy<br>☐ Chronic need for 24/7 care that is currently not in place in the home setting<br>☐<br>Other____			
Surgical/IR procedure required that cannot be performed in the home<br><br>If yes, select reason (if "other", select and describe):<br><br>☐ Surgical consultation required or high risk of being required<br>☐ Intraabdominal abscess requiring drainage (an example)<br>☐ Percutaneous nephrostomy tube placement (an example)<br>☐ Thoracentesis for parapneumonic effusion (an example)<br>☐ Significant surgical wound debridement<br>☐<br>Other____<br>__			
Clinical partnership with patient and family untenable to safely care for patient<br><br>If yes, select reason (if "other", select and describe):<br><br>☐ Severe altered mental status unable to be managed safely at home with family and home health aide (an example)<br>☐ Active substance abuse (an example)<br>☐ History of or high risk of noncompliance with primary treatment plan<br>☐<br>Other____<br>__			
Is the patient currently on hospice or have a life expectancy of <= 6 mo.?			

Social Stability Screening Tool

Patient Name: ____________________ Patient DOB: ___________ Program: ________________ Date of Screen: _______

Complete for ALL Programs

**Must be YES:**	**Yes**	**No**	**N/A**	**Comment**
Does the patient have a home recovery environment?				
Is the patient's home recovery environment in an eligible geography?				
Does the patient's home have water, electricity, bathroom, heat/ac, refrigerator?				
Does the patient feel safe at home?				
Are family/caregivers willing to participate, as needed, while the patient participates in the MH program?				
Is there a person in the home (patient or other) willing/able to take vital signs and answer phone calls?				
Is the patient willing to put pets in a secure area while MH affiliated staff are in the home?				
Is patient willing/able to participate in the MH program (this includes MH affiliated staff entering the home, and MH home equipment set-up in the home)?				
Is the patient/patient's HCP willing/able to sign consent?				

**Informative Questions:**	**Yes**	**No**	**N/A**	**Comment**
Does the patient have internet at home and a cell phone?				
Does the patient live in a stand-alone house or in a community setting? *Options: house, apartment, other*				
What floor does the patient live on?				
How many steps are there to enter the home?				
Is there an elevator at the home?				
Are there stairs within the home? *If yes-how many?*				
Does the patient live alone?				
Is there anyone who assists the patient with day to day life? *If yes-describe*				
Does the patient currently have any home services?				
Is the patient likely to require >4 hrs. of HHA support to fill ADL needs?				
Does the patient have mild, or greater, cognitive impairment?				
Has the patient experienced a fall within the last 6 mo.? *If yes, explain:*				
Is the patient homebound?				
Is the NIDA Quick Screen positive for the patient? *(see below for NIDA Quick Screen; if positive- explain)*				

Social Stability Screening Tool

Patient Name: ____________________ Patient DOB: ___________ Program: ________________ Date of Screen: _______

Is the NIDA Quick Screen positive for the anyone living in the patient's home? *(see below for NIDA Quick Screen; if positive- explain)*				
Is the patient's primary language English? *If no, what is the patient's primary language?*				

**Must be NO:**	**Yes**	**No**	**N/A**	**Comment**
Are there any behavioral diagnosis that would limit patient participation in the MH program?				
Does the patient require 1:1 aid with feeding or daily speech therapy?				
Does the patient require 1:1 aid with toileting and lack home assistance?				
Is the patient or caregiver unable to call for help or push the emergency help device?				
Is there active smoking in the home and need for home Oxygen?				
Are there unsecured firearms in the home?				
Does the patient exhibit any signs of neglect suggestive of an unsafe home?				
Is the patient deaf?				

**NIDA Quick Screen:**<br>In the past year, how many times have you used the following….						
Alcohol (for men, 5 or more drinks in a day; for women, 4 or more drinks in a day)	Never	Once or Twice	Monthly	Weekly	Almost Daily or Daily	Comment
Tobacco Products						
Prescription Drugs for Nonmedical Reasons						
Illegal Drugs (if in a state where marijuana is legal, specifically ask re: marijuana use)						

Social Stability Screening Tool

Patient Name: ____________________ Patient DOB: ___________ Program: ________________ Date of Screen: _______

Complete for SNF Substitution Program ONLY

Must be YES:	Yes	No	N/A	Comment
Has the patient been recommended for discharge to SNF?				
Are the patient/caregivers aware that HHA support provided by the MH program is temporary?				
Does the patient's home have sufficient space/appropriate condition for rehab needs?				
Are caregivers willing to aid the patient w/ADLs during the patient’s time in the MH program and post d/c from the MH program (if needed)?				

Must be NO:	Yes	No	N/A	Comment
Has the patient been previously recommended for long-term care?				
Does the patient live at a SNF/SAR/LTAC?				
Does the patient no longer prefer to live at home after the hospital/SNF stay?				
Has the patient required a 1:1 sitter in the last 48 hours?				
Has PT deemed the patient to be either a maximum assist or two assists in any functional category?				

Complete for ALL Programs

Must be YES:	Yes	No	N/A	Comment
Did the patient pass this screen?				
